# BT-DNBS: a novel cyanine-based turn-on fluorescent probe with large Stokes shift for sensitive and selective detection of biothiols in live-cell imaging†

**DOI:** 10.1039/d4ra07109c

**Published:** 2025-01-02

**Authors:** Shuai Zhang, Yoichiro Fujioka, Yusuke Ohba, Koji Yamada

**Affiliations:** a Graduate School of Environmental Science, Hokkaido University Japan zs929783910@eis.hokudai.ac.jp; b Department of Cell Physiology, Faculty of Medicine and Graduate School of Medicine, Hokkaido University Japan; c Division of Materials Science, Faculty of Environmental Earth Science, Hokkaido University Japan

## Abstract

Detecting biothiols like glutathione (GSH), homocysteine (Hcy), and cysteine (Cys) is key to understanding their roles in health and disease. We developed BT-DNBS, a cyanine-based turn-on fluorescent probe with a dinitrobenzenesulfonyl (DNBS) quencher group. Upon biothiol interaction, the quencher is cleaved, restoring fluorescence. The resulting probe BT-NH shows a maximum emission wavelength at 630 nm and a large Stokes shift (≈200 nm), enhancing detection accuracy. Low cytotoxicity and high time resolution make BT-DNBS suitable for live-cell imaging. Imaging of A431 cells confirmed intracellular biothiol detection, with NEM pre-treatment reducing fluorescence, verifying specificity. BT-DNBS holds promise for biomedical research, particularly in disease diagnostics.

## Abstract 2

BT-DNBS, a cyanine-based turn-on probe, features a large Stokes shift and low LOD for biothiol detection. Its fluorescence activation by biothiols allows sensitive imaging of thiol dynamics, with potential applications in biology and diagnostics.

## Graphical abstract

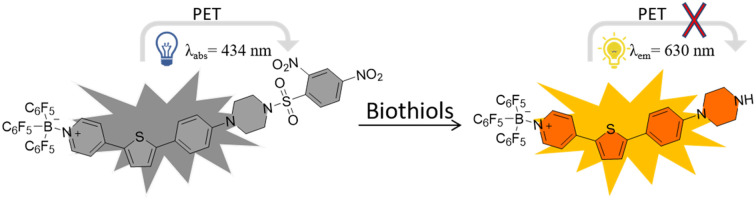

## 1. Introduction

GSH (glutathione), Hcy (homocysteine), and Cys (cysteine) play pivotal roles in human health. GSH (1–10 mM) acts as a potent antioxidant, protecting cells from oxidative stress and maintaining immune function.^1^ Elevated Hcy (12–15 μM) levels are linked to cardiovascular diseases,^2^ while Cys (30–200 μM) is crucial for protein synthesis and detoxification.^3^ Early detection of these biomarkers is crucial for preventing and treating related illnesses.^4^ Their assessment aids in identifying individuals at risk of cardiovascular diseases, neurodegenerative disorders, and other conditions, facilitating timely interventions.^5^ Thus, their evaluation holds significant promise for proactive healthcare management.

Traditional methods for biothiol detection often lack sensitivity, specificity, or compatibility with live-cell imaging.^6–8^ To address these challenges, fluorogenic techniques have emerged as powerful tools for biothiol detection, offering distinct advantages over conventional methods.^9^ These advantages include tunable optical properties, fast response times, high sensitivity (even at micromolar concentrations), cost-effectiveness and straightforward operational procedures.^10–12^ Fluorogenic techniques capitalize on the unique reactivity of biothiols with specific fluorogenic probes, resulting in enhanced fluorescence signals that enable sensitive and selective detection.^13^ Recent advancements in fluorogenic probe design and synthesis have further expanded the utility of these techniques for biothiol analysis in complex biological samples.^14–16^ However, most of those probes have small Stokes shift, typically less than 100 nm, resulting in overlapping excitation and emission spectra, which can hinder accurate detection.^17^ Additionally, many probes exhibit short emission wavelengths, limiting their utility for deep tissue imaging and *in vivo* applications.^18^ These challenges underscore the critical need for the development of new biothiol-targeting probes that can meet the rigorous demands of modern bioanalytical applications.

**Fig. 1:**
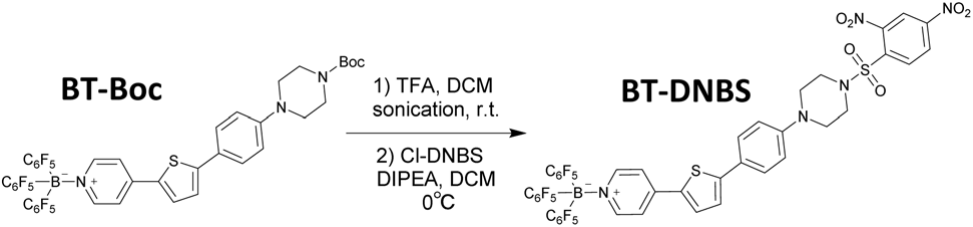
Simplified synthesis scheme for the fluorescent probe BT-DNBS.

**Fig. 2:**
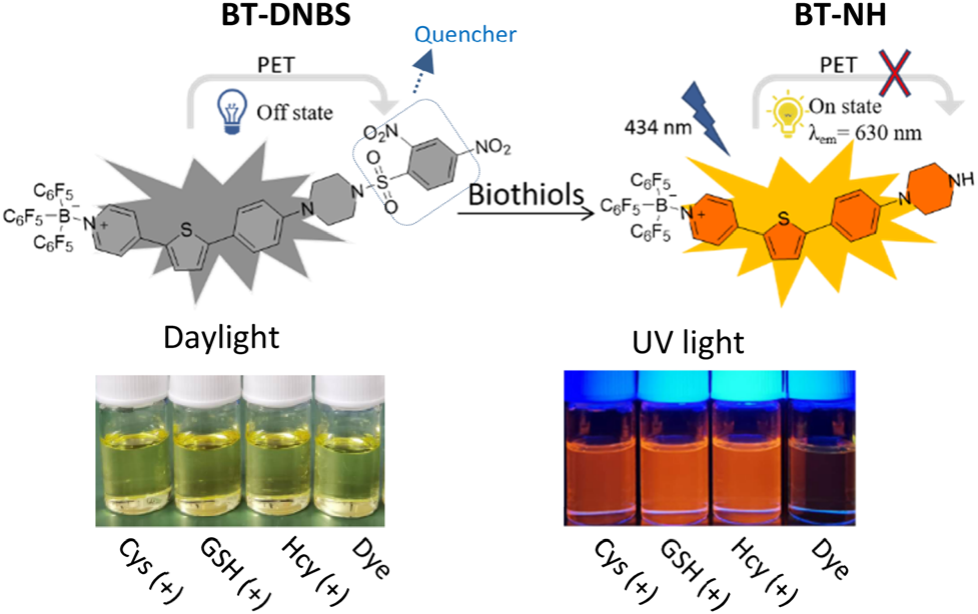
Proposed sensing mechanism of BT-DNBS for biothiol detection.

In response to the limitations of existing biothiol-targeting probes, a novel cyanine-based turn-on fluorescent probe, BT-DNBS, has been developed and applied in living cells, demonstrating significant advancements in biothiol detection. The simplified synthetic scheme is depicted in Fig. 1. The probe features a D–π–A framework (Fig. 2) with pyridinium as the electron-withdrawing group and piperazine as the electron-donating group. Additionally, the pyridinium moiety introduced by BCF [tris(pentafluorophenyl) borane, (B(C_6_F_5_)_3_)] enhances electron deficiency.^19^ DNBS serves as a commonly used fluorescence quenching moiety and reaction site for biotiols.^20–24^ After reacting with biothiols, the activated probe exhibits a maximum emission at 630 nm, resulting in a notably large Stokes shift of ≈200 nm. This substantial shift effectively minimizes spectral overlap and enhances detection accuracy. It also shows good selectivity over other analytes and linear relationship in concentration titration tests, along with lower cytotoxicity and stability in physiological pH conditions. Furthermore, we successfully applied this probe in living cells and identified its significant potential for broader applications in the field of biochemistry.

## 2. Results and discussion

The specific synthesis scheme for BT-DNBS is provided in the ESI.† All obtained compounds were characterized using ^1^H NMR spectroscopy. BT-Boc and BT-DNBS were characterized by ^1^H/^13^C NMR spectroscopy and ESI-MS spectrometry (ESI†).

### 2.1 Spectroscopic analysis of BT-DNBS towards biothiols

To optimize the experimental conditions for biothiol detection, the fluorescence intensity of BT-DNBS (10 μM) was evaluated in varying DMSO/PBS ratios (Fig. S7,† right). The results revealed a significant influence of the solvent composition on the fluorescence properties of the probe. Notably, the highest fluorescence intensity was achieved at a DMSO/PBS ratio of 9 : 1 (v/v). This ratio provides an optimal environment for both the solubility of the dye in the organic phase and the biological relevance of the aqueous phase, thus enhancing the fluorescence response upon biothiol interaction. As a result, all subsequent experiments involving biothiol detection were performed under this optimized solvent condition.

**Fig. 3:**
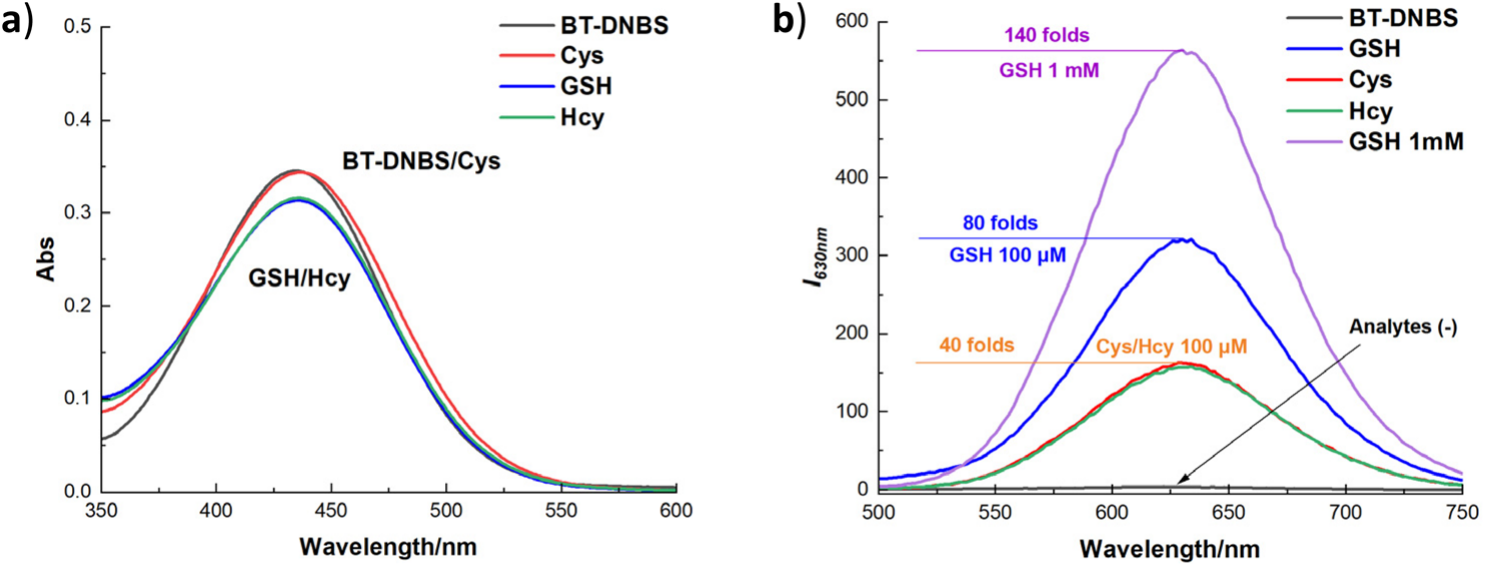
Spectral analysis of BT-DNBS towards biothiols. (a): Absorption spectra, (b): emission spectra of BT-DNBS (10 μM) in DMSO/PBS (9 : 1, v/v, pH 7.4) in the presence of biothiols (100 μM). Incubated for 20 min, *λ*_ex_ = 434 nm, *λ*_em_ = 630 nm.

Additionally, to assess the interaction of the fluorescent dye BT-DNBS with biothiols, absorption and emission spectra were recorded for BT-DNBS (10 μM) in the presence and absence of biothiols (100 μM). As shown in Fig. 3, the absorption spectra revealed minimal differences with and without biothiols, suggesting consistent dye behavior irrespective of biothiol presence. However, significant differences were observed in the emission spectra. In the absence of biothiols, only weak fluorescence was detected, implying effective fluorescence quenching by the DNBS motif. In contrast, the introduction of biothiols resulted in a pronounced turn-on fluorescence response, highlighting a notably strong and observable orange fluorescence signal. Moreover, the fluorescence intensity increased by up to 40-fold for Cys/Hcy (100 μM), 80-fold for GSH (100 μM), and notably, up to 140-fold for 1 mM GSH, demonstrating a highly sensitive response to biothiols, especially for GSH. A plausible mechanism for the stronger fluorescence response of GSH compared to Cys and Hcy may involve the formation of an intermediate through an S–C bond between GSH and the DNBS group. The larger, flexible structure of GSH allows for effective intramolecular hydrogen bonding, facilitating hydrogen transfer and promoting the cleavage of the sulfonate group, which releases the fluorescent moiety more readily. Additionally, concentration titration experiments ranging from pure BT-DNBS to varying concentrations of biothiols (0–1000 μM) revealed a good linear relationship (ESI†). Furthermore, ranging from 0–30 μM of biothiols, the limit of detection (LOD) was calculated as 83 nM for GSH, 49 nM for Cys, and 80 nM for Hcy, highlighting the probe's potential for sensitive biothiol detection and quantification. After reacting with biothiols, the activated probe BT-NH demonstrated a maximum emission wavelength at 630 nm, resulting in a substantial Stokes shift (≈200 nm) that effectively minimizes spectral overlap and enhances detection accuracy. To better contextualize the advantages of our probe, we have provided a comparison table (Table S2, ESI†) summarizing reported fluorescent probes containing the DNBS group as a receptor unit. Our probe demonstrates a significantly larger bathochromic shift and a lower LOD (83 nm for GSH, 49 nm for Cys and 80 nm for Hcy) relative to the reported probes. These properties contribute to the probe's enhanced performance in live-cell imaging applications, making it a valuable tool for sensitive and selective detection of biothiols.

### 2.2 Selectivity analysis of BT-DNBS towards analytes

**Fig. 4:**
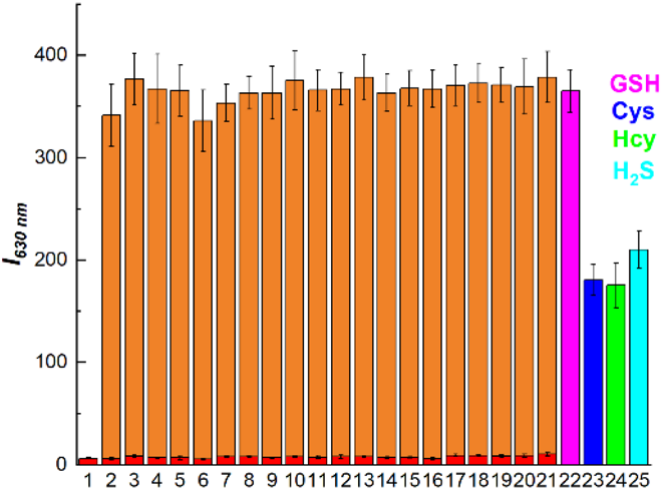
Analysis of fluorescence intensity response of BT-DNBS to different analytes. BT-DNBS (10 μM) in DMSO/PBS (9 : 1, v/v, pH 7.4) in the presence of various analytes (100 μM). Incubated for 20 min, *λ*_ex_ = 434 nm, *λ*_em_ = 630 nm. (1): BT-DNBS (control); analytes included (2–21): Arg, Gly, His, Leu, Lys, Met, Phe, Pro, Ser, Thr, Cl^−^, Br^−^, NO_2_^−^, HCO_3_^−^, SO_3_^2−^, SO_4_^2−^, Ca^2+^, Fe^2+^, Mg^2+^, Vc; (22–25), GSH, Cys, Hcy, H_2_S. Orange bar: fluorescence response measured by first adding interfering species (2–21), followed by the addition of GSH.

To validate the sensitivity and specificity of BT-DNBS in identifying biothiols, a range of analytes were employed and analyzed. As illustrated in Fig. 4, the fluorescence intensity of BT-DNBS remained relatively unchanged upon the addition of various analysts (including Arg, Gly, His, Leu, Met, Phe, Pro, Ser, Thr, Cl^−^, Br^−^, NO_2_^−^, HCO_3_^−^, SO_3_^2−^, SO_4_^2−^, Ca^2+^, Fe^2+^, Mg^2+^, Na^+^, Vc) compared to the BT-DNBS alone (Fig. 4). However, notable fluorescence responses were observed in the presence of biothiols, suggesting that BT-DNBS exhibited strong sensing capabilities toward biothiols compared to other analytes. The high selectivity of BT-DNBS for biothiols can be attributed to the specific interaction between the DNBS moiety and thiol groups. Interestingly, the probe also responded to H_2_S, with a fluorescence intensity similar to that observed for Cys and Hcy. This could also be attributed to the nucleophilic properties of H_2_S, which allows it to interact with the DNBS moiety of BT-DNBS, leading to fluorescence activation. The negligible interference from other common biological and chemical analytes highlight the probe's potential for accurate biothiol detection in complex biological environments. These findings confirm that BT-DNBS is a highly selective and sensitive probe for biothiol detection, making it suitable for applications in bioanalytical chemistry and medical diagnostics.

### 2.3 Time & pH-dependent spectroscopic analysis towards biothiols

**Fig. 5:**
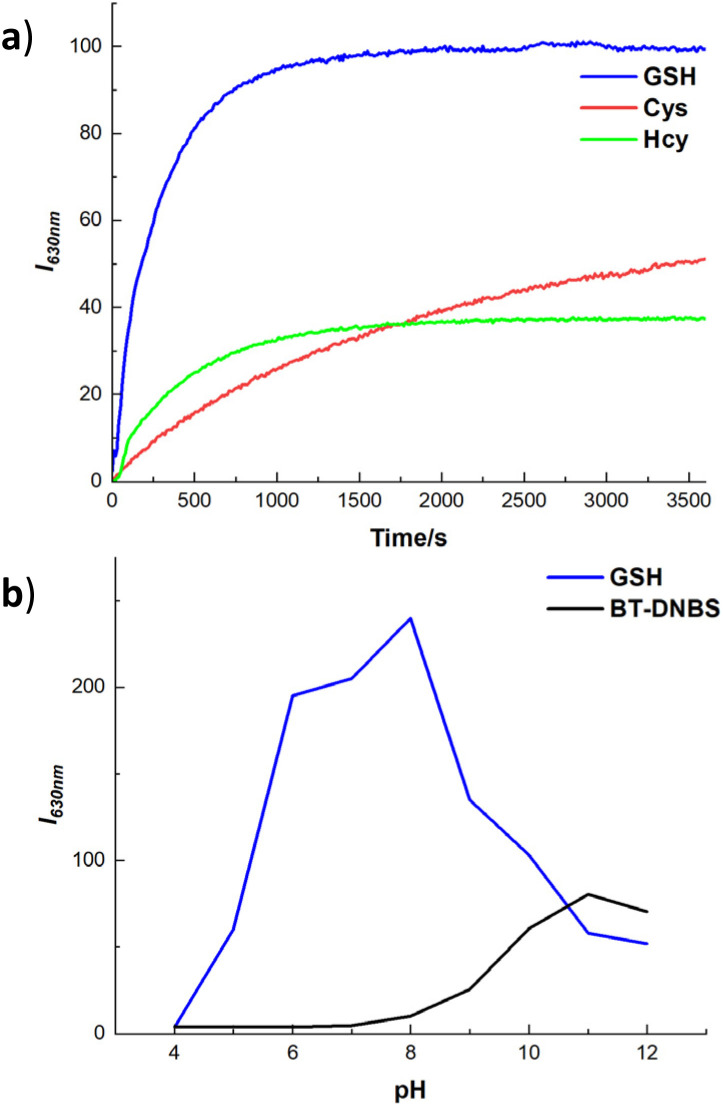
Analysis of response time and pH stability of BT-DNBS towards biothiols. (a): Time-dependent fluorescence intensity response of BT-DNBS (10 μM, DMSO/PBS = 9 : 1, v/v, pH 7.4) in the presence of biothiols (100 μM), GSH (blue), Cys (red), and Hcy (green). *λ*_ex_ = 434 nm, *λ*_em_ = 630 nm. (b): pH-dependent fluorescence intensity response of BT-DNBS (10 μM, DMSO/PBS = 9 : 1, v/v) from pH 4 to 12 without analytes (black) and in the presence of GSH (100 μM, blue). Incubated for 20 min, *λ*_ex_ = 434 nm, *λ*_em_ = 630 nm.

To evaluate the temporal and pH response of BT-DNBS towards biothiols, we conducted a time- and pH-dependent fluorescence intensity analysis. This analysis aimed to determine the kinetics, sensitivity, and pH stability of BT-DNBS when exposed to different biothiols over time and across various pH levels. The fluorescence intensity was monitored at regular intervals to observe the dynamic interaction between BT-DNBS and the target biothiols (GSH, Cys, and Hcy). As depicted in Fig. 5(a), the fluorescence intensity exhibited a rapid increase within the initial 500 s, reaching near-maximum intensity at approximately 1200 s. Subsequently, the fluorescence intensity stabilized for the remaining 1 hour observation period. This swift and consistent response suggests that BT-DNBS possesses high efficiency in detecting biothiols, rendering it advantageous for real-time monitoring applications. Furthermore, it is noteworthy that the response time of BT-DNBS is shorter compared to many biothiol-targeted fluorogenic probes.^25–27^ The fluorescence intensity changes of BT-DNBS in response to pH variation were investigated across a range from pH 4 to pH 12, as shown in Fig. 5(b). Notably, when BT-DNBS was tested without biothiols, it exhibited stable weak fluorescence intensity between pH 6 and 8. This stability suggests that BT-DNBS remained in a fluorescence-off state under these pH conditions. However, in the presence of GSH, a significant fluorescence-on state was observed within the same pH range of 6 to 8. This indicates that the addition of GSH induced a notable increase in fluorescence intensity, demonstrating the effective response of BT-DNBS to biothiols under physiological pH conditions. However, as the pH increases beyond 8, the fluorescence intensity further increases, probably due to the deprotonation of the DNBS group, which reduces the non-radiative decay pathways and promotes more efficient fluorescence emission. These findings underscore the pH-responsive behavior of BT-DNBS and its ability to detect biothiols under specific pH environments, making it a promising candidate for applications in physiological and biological systems.

### 2.4 Sensing mechanism

**Fig. 6:**
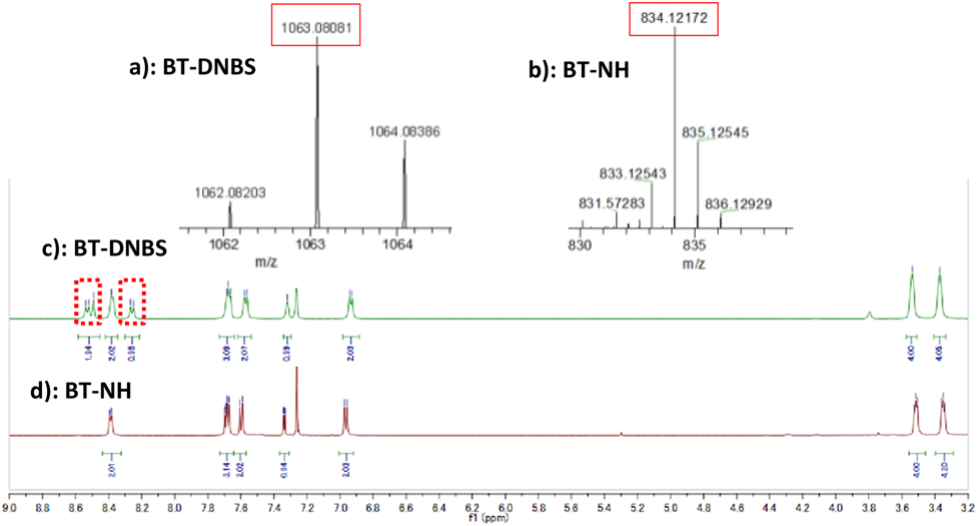
^1^H NMR and ESI-mass spectra confirming the proposed sensing mechanism of BT-DNBS. (a): ESI-mass spectrum of BT-DNBS. [M]^+^ calculated: 1063.0787; found: 1063.0808. (b): ESI-mass spectrum of isolated BT-NH after BT-DNBS reacted with GSH. [M + H]^+^ calculated: 834.1220; found: 834.1217. (c): ^1^H NMR spectrum of BT-DNBS. (d): ^1^H NMR spectrum of isolated BT-NH after BT-DNBS reacted with GSH.

Previous studies have demonstrated that probes incorporating DNBS group as a recognition site undergo nucleophilic aromatic substitution reactions with biothiols.^28,29^ This reaction mechanism involves the removal of the DNBS group, which suppresses the photo-induced electron transfer (PET), restoring the inherent fluorescence of BT-NH. In the context of BT-DNBS, upon interaction with biothiols such as GSH, Cys, and Hcy, the DNBS group, initially acting as a fluorescence quencher by the intramolecular PET, is cleaved, thereby resulting in a pronounced fluorescence enhancement. To validate the sensing mechanism, BT-DNBS and isolated product BT-NH were analyzed using ^1^H NMR and ESI-mass spectrometry, as depicted in Fig. 6. The ^1^H NMR spectra of the isolated reaction product BT-NH showed the disappearance of peaks corresponding to the cleaved DNBS group. Additionally, ESI-mass spectrometry confirmed the presence of a new peak corresponding to BT-NH. These findings provide compelling evidence for the nucleophilic substitution reaction and the subsequent formation of the fluorescent BT-NH product, corroborating the proposed mechanism.

### 2.5 Density functional theory (DFT) calculation

**Fig. 7:**
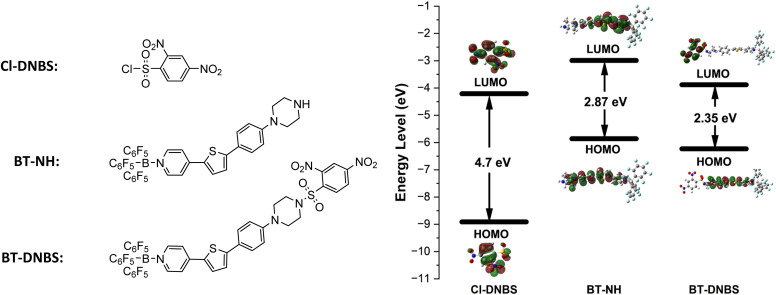
Density Functional Theory (DFT) optimized structures and frontier molecular orbitals (MOs) of Cl-DNBS, BT-NH, and BT-DNBS. Calculations were performed based on ground state geometries using the B3LYP functional and the 6-311G(d,p) basis set with the Gaussian 09W program.

To better understand the fluorescence turn-on mechanism of BT-DNBS from the perspective of frontier molecular orbitals, DFT calculations and structure optimizations were performed for Cl-DNBS, BT-NH, and BT-DNBS using the B3LYP functional and the 6-311G(d,p) basis set, as implemented in the Gaussian 09 program. As shown in Fig. 7, the results indicate that the LUMO (Lowest Unoccupied Molecular Orbital) energy level of Cl-DNBS (−4.21 eV) is positioned between the HOMO (−5.86 eV) (Highest Occupied Molecular Orbital) and LUMO (−2.99 eV) of BT-NH. This suggests that the PET (photoinduced electron transfer) process from excited BT-NH to Cl-DNBS is thermodynamically favorable. Upon reaction of BT-DNBS with biothiols, the N–S bond between the piperazine and DNBS is cleaved, which interrupts the PET process, resulting in the restoration of fluorescence. Moreover, the π-electrons of BT-NH are primarily distributed in both the HOMO and LUMO orbitals, whereas for BT-DNBS, the π-electrons in the HOMO are mainly localized on the dye moiety, and the π-electrons in the LUMO are predominantly distributed on the DNBS moiety. This distribution further supports that the PET process from BT-NH to Cl-DNBS effectively quenches the dye's fluorescence.

### 2.6 Live-cell imaging

**Fig. 8:**
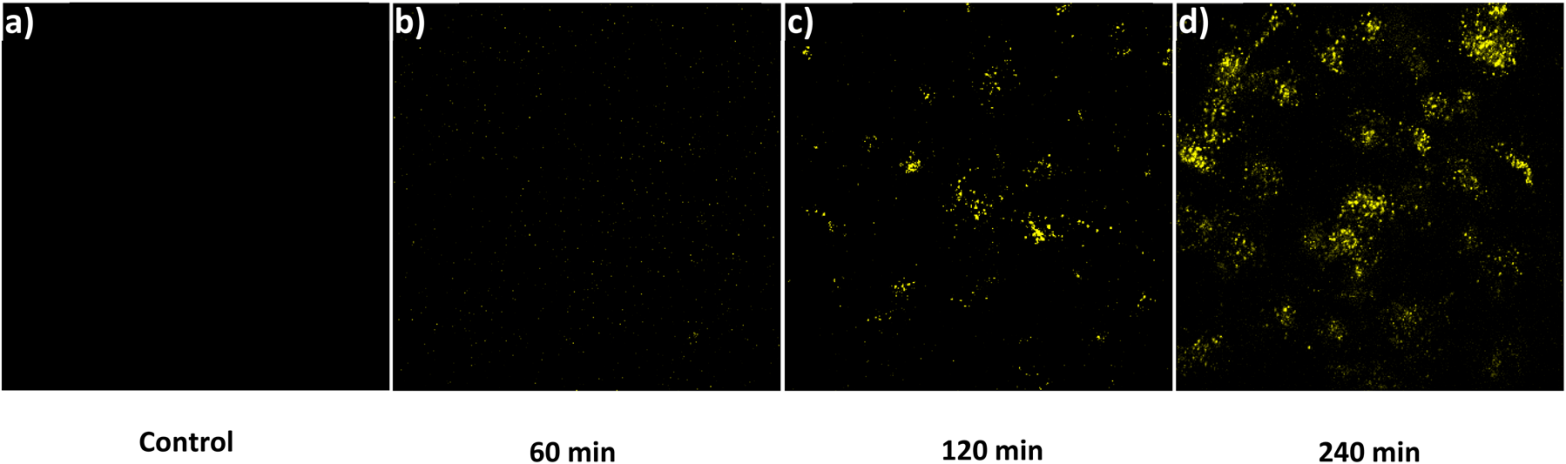
Fluorescent images. (a): Control group, untreated A431 cells. (b–d): Cells incubated with BT-DNBS (10 μM) for 60 minutes (b), 120 minutes (c), 240 minutes (d). *λ*_ex_ = 434 nm, *λ*_em_ = 630 nm.

Before evaluating the imaging capabilities of BT-DNBS, a cell viability assay was performed to assess its cytotoxicity using CCK-8. A431 cells were treated with varying concentrations of BT-DNBS (0, 1, 10, 100 μM) for 24 hours. The results, depicted in Fig. S8 (ESI†), confirmed the low cytotoxicity of BT-DNBS at an applied concentration of 10 μM for cell imaging applications. Following this, we conducted fluorescence imaging experiments using A431 cells to evaluate the practical applicability of BT-DNBS for live-cell imaging. The cells were incubated with BT-DNBS (10 μM) for different time periods (60, 120, and 240 minutes) at 37 °C. Confocal images were acquired using a spinning disk confocal microscopy. As depicted in Fig. 8(a), the untreated cells (control group) exhibited negligible fluorescence, indicating lower inherent autofluorescence. In contrast, the BT-DNBS-treated cells showed a time-dependent increase in fluorescence intensity: weak fluorescence after 60 minutes (Fig. 8(b)), stronger fluorescence after 120 minutes (Fig. 8(c)), and even stronger fluorescence after 240 minutes (Fig. 8(d)).

**Fig. 9:**
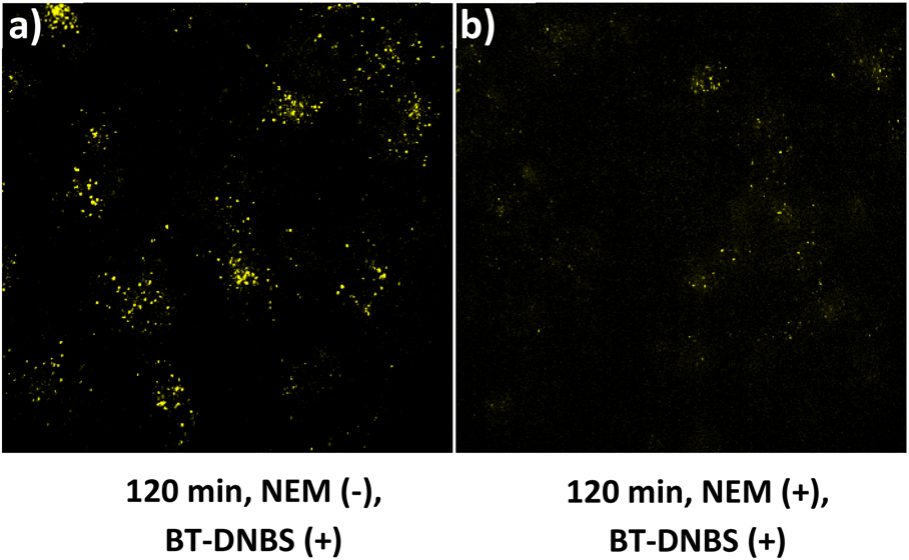
Specificity analysis of biothiols. (a): Cells incubated with BT-DNBS (10 μM) for 120 minutes without NEM pre-treatment. (b): Cells were pre-treated with NEM (50 μM) for 60 minutes, followed by incubation with BT-DNBS (10 μM) for 120 minutes. *λ*_ex_ = 434 nm, *λ*_em_ = 630 nm.

To confirm that the fluorescence observed upon BT-DNBS treatment is due to its specific reaction with intracellular biothiols, we conducted NEM (*N*-ethylmaleimide) inhibition experiments. NEM is a known thiol-blocking agent,^30^ and pre-treatment with NEM could weaken fluorescence if BT-DNBS specifically reacts with biothiols. A431 cells were incubated with BT-DNBS (10 μM) for 120 minutes under two conditions: in the absence of NEM (Fig. 9(a)) and in the presence of NEM (50 μM pre-treatment for 60 minutes) (Fig. 9(b)). As expected, cells treated with BT-DNBS alone showed visible fluorescence. In contrast, cells pre-treated with NEM showed weaker fluorescence after BT-DNBS incubation. These results demonstrate that the fluorescence activation of BT-DNBS is specifically triggered by its interaction with biothiols.

## 3. Conclusion

In this study, we developed and assessed a novel cyanine-based fluorescent probe, BT-DNBS, for biothiol detection. BT-DNBS showed significant fluorescence enhancement upon reacting with biothiols due to a nucleophilic aromatic substitution reaction, confirmed by ^1^H NMR and ESI-mass spectrometry. The probe exhibited high sensitivity with detection limits of 83 nM for GSH, 49 nM for Cys, and 80 nM for Hcy. Upon activation by biothiols, the resulting probe BT-NH exhibited a maximum emission wavelength of 630 nm, with a significant Stokes shift of ≈200 nm. Selectivity tests confirmed its specificity for biothiols over other analytes. Time- and pH-dependent studies demonstrated its stability and rapid response in physiological conditions. Cytotoxicity assay indicated low toxicity of BT-DNBS at relevant concentrations, supporting its suitability for live-cell applications. Live-cell imaging showed that BT-DNBS could detect biothiols within cells, primarily localizing in the cytoplasm. Additionally, NEM inhibition experiments confirmed the biothiol-specific activation of fluorescence, further supporting the selectivity of the probe. These results suggest that BT-DNBS may serve as a valuable tool for imaging biothiol fluctuations in live cells, with potential applications in disease diagnostics and studies of cellular redox biology. However, it is essential to note that further investigation is required to assess its viability for time-lapse imaging, particularly in determining whether the dye remains stable under continuous excitation light.

## 4. Experimental

The detailed information of materials, reagents, instruments, synthesis, spectroscopic analysis, cell culture, cytotoxicity assay, fluorescence imaging experiments are provided in the ESI.†

### 4.1 Synthesis of BT-DNBS

BT-Boc (100 mg, 0.11 mmol) was dissolved in dichloromethane (3 mL) in a dried flask. Trifluoroacetic acid (0.6 mL) was subsequently added dropwise to the flask, and the resulting mixture was sonicated for 1 hour at room temperature. The solvent was then evaporated, and the residue underwent vacuum drying for 15 hours. Following this, the resultant material was dissolved in dichloromethane (3 mL), and *N*,*N*-diisopropylethylamine (57 μL, 0.33 mmol) was added, maintaining the reaction at 0 °C. A mixture of dinitrobenzenesulfonyl chloride (59 mg, 0.22 mmol) and dichloromethane (2 mL) was then slowly added to the reaction system and stirred for 30 minutes. The reaction mixture was subsequently allowed to return to room temperature and stirred for an additional 16 hours. Upon completion of the reaction, as monitored by TLC (hexane : ethyl acetate = 1 : 1), the mixture was subjected to purification *via* silica gel chromatography (hexane : ethyl acetate = 2 : 1) resulting in the isolation of compound BT-DNBS as a brown solid. (104 mg, 89%).

^1^H NMR (500 MHz, CDCl_3_, TMS, r.t.): *δ* 8.53 (dd, *J* = 8.5, 2.0 Hz, 1H), 8.49 (d, *J* = 2.0 Hz, 1H) 8.38 (d, *J* = 6.5 Hz, 2H), 8.26 (d, *J* = 9.0 Hz, 1H), 7.68 (d, *J* = 4.0 Hz, 1H), 7.67 (d, *J* = 7.5 Hz, 2H), 7.57 (d, *J* = 9.0 Hz, 2H), 7.32 (d, *J* = 4.0 Hz, 1H), 6.93 (d, *J* = 9.0 Hz, 2H), 3.54 (t, *J* = 5.0 Hz, 4H), 3.37 (t, *J* = 5.0 Hz, 4H). ^13^C NMR (CDCl_3_, 125 MHz, r.t.): *δ* = 152.24, 151.56, 150.49, 148.94, 147.54, 146.84, 137.57, 135.22, 133.16, 131.46, 127.79, 126.59, 125.32, 124.55, 120.19, 117.01, 48.66, 45.77.

ESI-MS (*m*/*z*): [M]^+^ calcd for C_43_H_21_BF_15_N_5_O_6_S_2_^+^: 1063.0787; Found: 1063.0808.

## Author contributions

Shuai Zhang: conceptualization, methodology, data curation, formal analysis, investigation, writing—original draft preparation, and project administration. Yoichiro Fujioka and Yusuke Ohba: provided technical support and resources for cell imaging experiments. Koji Yamada: supervision, project administration, funding acquisition, resources, reviewing the manuscript, corresponding author responsibilities. All authors have read and approved the final manuscript.

## Conflicts of interest

There are no conflicts to declare.

## Supplementary Material

RA-015-D4RA07109C-s001

## Data Availability

The authors confirm that the data supporting the findings of this study are available within the article [and/or its ESI materials†].
