# Supplementary material for: BT-DNBS: a novel cyanine-based turn-on fluorescent probe with large Stokes shift for sensitive and selective detection of biothiols in live-cell imaging
Source: RSC Adv. 2025 Jan 2;15(1):135–41. doi: 10.1039/d4ra07109c (PMC11694346; doi:10.1039/d4ra07109c)
Supplement: RA-015-D4RA07109C-s001 [file RA-015-D4RA07109C-s001.pdf]

Supplementary Information (SI) for RSC Advances.
This journal is © The Royal Society of Chemistry 2024

Supporting Information

BT-DNBS: A Novel Cyanine-Based Turn-On Fluorescent Probe with Large Stokes Shift for Sensitive and Selective Detection of Biothiols in Live-Cell Imaging

Shuai Zhang,[a] Yoichiro Fujioka,[b] Yusuke Ohba[b] and Koji Yamada*[c]

a. Graduate School of Environmental Science, Hokkaido University, Sapporo 060-0810, Japan.

E-mail: zs929783910@eis.hokudai.ac.jp

b. Department of Cell Physiology, Faculty of Medicine and Graduate School of Medicine, Hokkaido University.

c. Division of Materials Science, Faculty of Environmental Science, Hokkaido University.

1. Synthesis Scheme of BT-DNBS

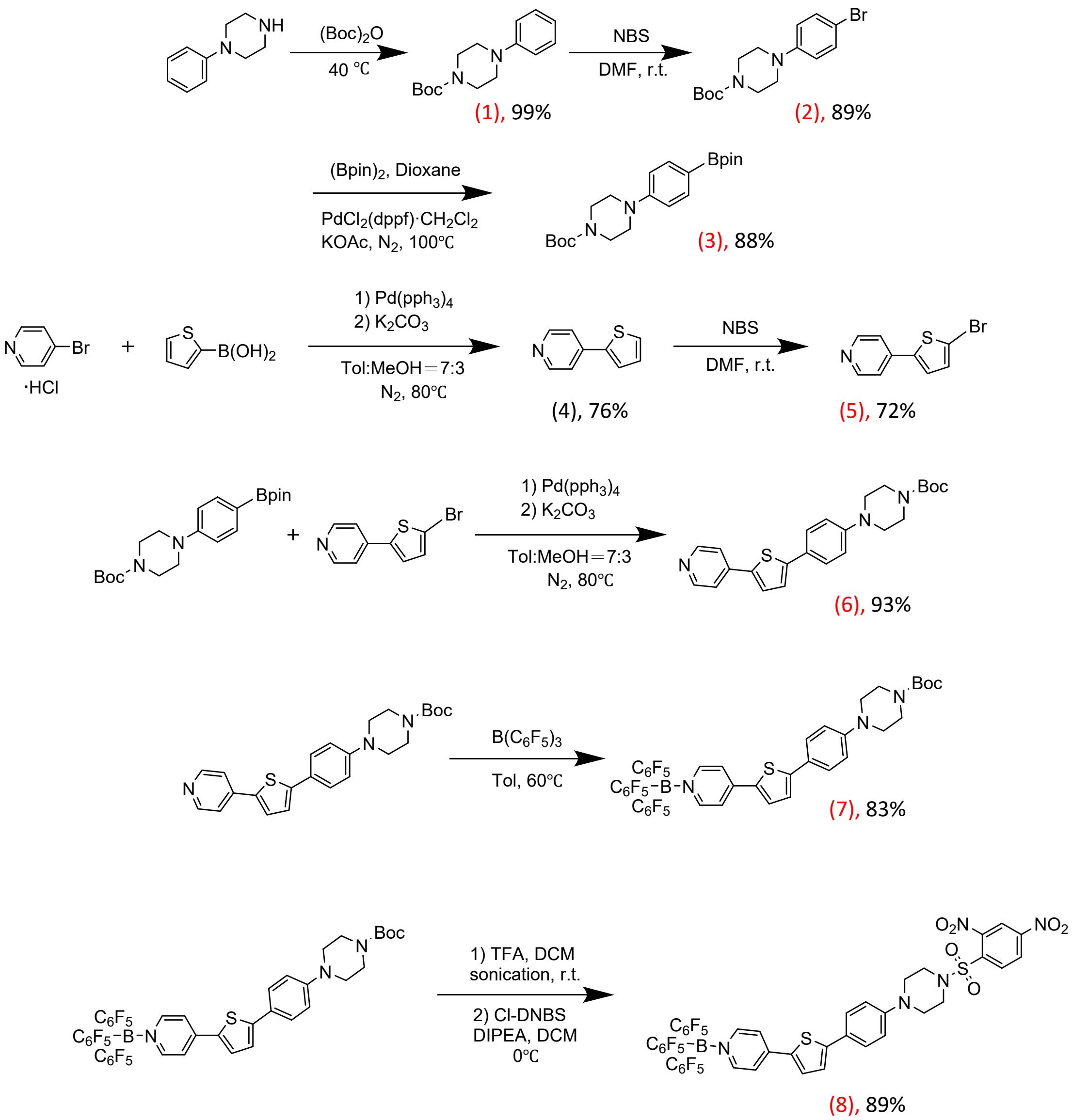

The yields are expressed as percentages.

Materials, Instruments and Methods

Unless otherwise noted, all solvents and chemicals were of reagent grade, with purity ranging from 95% to 99%. Solvents used for spectral analysis were of high purity, at 99.8%. All chemicals and solvents were purchased from Sigma-Aldrich, Kanto Chemical, Tokyo Chemical Industry, and Wako Pure Chemical Industries, and were used without further purification. Reaction processes were monitored by thin-layer chromatography (TLC) on pre-coated silica gel plates (silica gel 60, 0.25, F-254), visualized under UV light and/or by chromogenic reaction. Silica gel 60 (230-400 mesh) was used for column chromatography.

^{1}H-NMR spectra were recorded on JEOL JNN-EX 400 MHz or JEOL JNM-ECZR 500 MHz spectrometers at room temperature. ^{13}C NMR spectra were recorded at 125 MHz. Chemical shifts (δ) were reported as ppm in $CDCl_3$ or DMSO-d_6, with TMS as the internal standard. Coupling constants (*J*) were expressed in hertz (Hz). Mass spectra were carried out under ESI conditions (Thermo Scientific Exactive, Thermo Fisher Scientific K.K., Tokyo, Japan, and JMS-T100LP (JEOL) USA Inc.).

Tert-butyl 4-phenylpiperazine-1-carboxylate (1).

1-Phenylpiperazine (6.15 g, 37.9 mmol) and di-*tert*-butyl pyrocarbonate (9.10 g, 41.7 mmol) were combined and subjected to sonication for 40 minutes in a water bath at 40 ℃. Upon the completion of the reaction, as monitored by thin-layer chromatography (TLC) (R_f=0.3, hexane: ethyl acetate=5:1). the mixture was cooled down and transitioned into a transparent solid compound **1**. (11 g, 99%).

^{1}H NMR (400 MHz, $CDCl_3$, TMS, r.t.): δ 7.31-7.25 (m, 2H), 6.99-6.85 (m, 3H), 3.58 (t, *J* = 5.2 Hz, 4H), 3.13 (t, *J* = 5.2 Hz, 4H), 1.48 (s, 9H).

Tert-butyl 4-(4-bromophenyl) piperazine-1-carboxylate (2).

N-Bromosuccinamide (2.39 g, 12.58 mmol) was dissolved in 15 mL of anhydrous DMF and added dropwise to a stirred solution of compound **1** (3 g, 11.43 mmol) in 15 mL of anhydrous DMF, which has been shielded from light with aluminum foil at 0 ℃. After the addition of NBS, the reaction mixture was then allowed to reach room temperature and stirred for 24 hours. Upon completion of the reaction, as confirmed by TLC (R_f=0.4, hexane: ethyl acetate = 2:1), ice-water (10 mL) was then introduced, leading to the precipitation of the product. The resulting precipitate was isolated by filtration, and the crude product was subsequently purified by silica gel column chromatography (hexane: ethyl acetate = 15:1) and evaporated to yield a white solid **2**. (3.47g, 89%)

^{1}H NMR (400 MHz, $CDCl_3$, TMS, r.t.): δ 7.35 (d, *J* = 8.8 Hz, 2H), 6.79 (d, *J* = 8.8 Hz, 2H), 3.57 (t, *J* = 5.2 Hz, 4H), 3.10 (t, *J* = 5.2 Hz, 4H), 1.48 (s, 9H).

Tert-butyl 4-(4-(4,4,5,5-tetramethyl-1,3,2-dioxaborolan-2-yl) phenyl) piperazine-1-carboxylate (3).

In a dry two-necked flask, compound **2** (1.5 g, 4.4 mmol), KOAc (518 mg, 5.27 mmol), and $PdCl_2(dppf)\cdot CH_2Cl_2$ (72 mg, 0.09 mmol) were dissolved in 1,4-dioxane (20 mL). The resulting mixture was purged with N_2 three times and refluxed at 100 ℃ for 5 minutes. Subsequently, a mixture of bis(pinacolato)diboron (1.34 g, 5.27 mmol) and 1,4-dioxane (15 mL) was added slowly, and the reaction stirred for 14 hours. After the completion of the reaction, as confirmed by TLC (R_f=0.2, hexane: ethyl acetate = 9:1), the mixture was then extracted with ethyl acetate, washed with water and brine, and dried over anhydrous Na_2SO_4 successively. The drying agent was then removed by filtration. The crude product was further purified by silica gel chromatography (hexane: ethyl acetate = 9:1) and evaporated to yield a white solid **3**. (1.5 g, 88%)

^{1}H NMR (400 MHz, $CDCl_3$, TMS, r.t.): δ 7.52 (d, *J* = 8.8 Hz, 2H), 6.68 (d, *J* = 8.8 Hz, 2H), 3.56 (t, *J* = 5.2 Hz, 4H), 3.10 (t, *J* = 5.2 Hz, 4H), 1.48 (s, 9H) 1.25 (s, 12H).

4-(thiophen-2-yl) pyridine (4).

In a dried flask, 4-Bromopyridine hydrochloride (2 g, 10.28 mmol), 2-thienylboronic acid (1.32 g, 10.28 mmol), K_2CO_3 (2.84 g, 20.56 mmol), and Pd $(PPh_3)_4$ (593 mg, 0.50 mmol) were dissolved in a mixed solvent (Toluene/MeOH = 7/3, 50 mL). The reaction proceeded under a nitrogen atmosphere at 80 ℃ for 18 hours, monitored by TLC (R_f=0.2, hexane: ethyl acetate = 3:2). Upon completion, the reaction mixture was quenched with ice-water and extracted with ethyl acetate. The combined organic extracts were concentrated, resulting in a 10% volume of the initial amount. The concentrated organic phase underwent extraction with 0.5 N HCl (5 × 20 mL), followed by neutralization using a saturated $NaHCO_3$ solution until no more gas bubbles were produced. Then the mixture underwent an additional extraction with ethyl acetate, followed by drying over anhydrous Na_2SO_4. Concentration of the organic phase yielded a gray solid product **4** (1,27 g, 76%).

^{1}H NMR (400 MHz, $CDCl_3$, TMS, r.t.): δ 8.60 (d, *J* = 4.8 Hz, 2H), 7.50 (dd, *J* = 3.6 Hz, 1.2 Hz, 1H), 7.48 (dd, *J* = 4.8 Hz, 2.0 Hz, 2H), 7.42 (dd, *J* = 5.2 Hz, 1.2 Hz, 1H), 7.14 (dd, *J* = 5.2 Hz, 1.2 Hz, 1H).

4-(5-bromothiophen-2-yl) pyridine (5).

Compound 4 (990 mg, 6.14 mmol) was dissolved in dehydrated DMF 20 mL in a dried flask at 0 ℃. A mixture of N-Bromosuccinamide (1.31 g, 7.37 mmol) and DMF 5 mL was then added dropwise to the reaction system under darkness. After 30 minutes, the reaction mixture was further stirred for 18 hours at room temperature. Upon completion of the reaction, as monitored by TLC (R_f=0.15, hexane: ethyl acetate = 5:1), the mixture was extracted with ethyl acetate, washed by $NaHCO_{3(aq)}$, $NaCl_{(aq)}$, dried over anhydrous Na_2SO_4. The drying agent was then removed by filtration. The crude product was subsequently purified by silica gel chromatography (hexane: ethyl acetate = 5:1) and evaporated to yield a brown solid **5**. (1.06 g, 72%)

^{1}H NMR (400 MHz, $CDCl_3$, TMS, r.t.): δ 8.61 (br, 2H), 7.39 (d, *J* = 5.6 Hz, 2H), 7.27 (d, *J* = 1.6 Hz, 1H), 7.10 (d, *J* = 4.0 Hz, 1H).

Tert-butyl 4-(4-(5-(pyridin-4-yl) thiophen-2-yl) phenyl) piperazine-1-carboxylate (6).

In a dried flask, compound **3** (888 mg, 2.29 mmol), compound **5** (500 mg, 2.08 mmol), K_2CO_3 (575 mg, 4.16 mmol), and Pd $(PPh_3)_4$ (170 mg, 0.14 mmol) were dissolved in a mixed solvent (Toluene/MeOH = 7/3, 25 mL). The reaction proceeded under a nitrogen atmosphere at 80 ℃ for 12 hours, monitored by TLC (R_f=0.2, hexane: ethyl acetate = 3:2). Upon completion, the reaction mixture was quenched with ice-water and extracted with ethyl acetate, washed with H_2O, and brine, then dried over anhydrous Na_2SO_4. The drying agent was then removed by filtration. The crude product was subsequently purified by silica gel chromatography (hexane: ethyl acetate = 1:1) and evaporated to yield a yellow powder **6**. (815 mg, 93%)

^{1}H NMR (400 MHz, $CDCl_3$, TMS, r.t.): δ 8.57 (br, 2H), 7.55 (d, *J* = 8.8 Hz, 2H), 7.46 (m, 3H), 7.21 (d, *J* = 4.0 Hz, 1H), 6.93 (d, *J* = 8.8 Hz, 2H), 3.60 (t, *J* = 5.2 Hz, 4H), 3.20 (t, *J* = 5.2 Hz, 4H), 1.49 (s, 9H).

ESI-Mass (m/z): $[M+H]^+$ calcd for $C_{24}H_{28}N_3O_2S^+$: 422.19; Found: 422.19.

(4-(5-(4-(4-(*tert*-butoxycarbonyl) piperazin-1-yl) phenyl) thiophen-2-yl)-1λ^4-pyridin-1-yl) tris(pentafluorophenyl) borate (7), BT-Boc

In a dried flask, compound **6** (200 mg, 0.48 mmol) and tris(pentafluorophenyl)borane (270 mg, 0.53 mmol) were dissolved in toluene (5 mL) at 60 ℃ under nitrogen condition and stirred for 24 hours. Following the completion of the reaction, as monitored by TLC (R_f=0.5, hexane: ethyl acetate = 1:1), the reaction mixture was extracted with ethyl acetate, subjected to $NaHCO_{3(aq)}$ and brine washes, dried over anhydrous Na_2SO_4. The drying agent was then removed by filtration. The crude product was further purified via silica gel chromatography (hexane: ethyl acetate = 5:1) and evaporated, resulting in the isolation of compound **7** as a yellow solid. (371 mg, 83%)

^{1}H NMR (500 MHz, $CDCl_3$, TMS, r.t.): δ 8.36 (d, *J* = 6.0 Hz, 2H), 7.68 (d, *J* = 4.0 Hz, 1H), 7.66 (d, *J* = 7.5 Hz, 2H), 7.57 (d, *J* = 9.0 Hz, 2H), 7.31 (d, *J* = 4.0 Hz, 1H), 6.94 (d, *J* = 9.0 Hz, 2H), 3.60 (t, *J* = 5.0 Hz, 4H), 3.25 (t, *J* = 5.0 Hz, 4H), 1.49 (s, 9H).

ESI-Mass (m/z): $[M]^+$ calcd for $C_{42}H_{27}BF_{15}N_3O_2S^+$: 933.17; Found: 933.17.

(5-(5-(4-(4-((2,4-dinitrophenyl) sulfonyl) piperazin-1-yl) phenyl) thiophen-2-yl)-1λ^4-pyridin-1-yl) tris(pentafluoro - phenyl)borate (8), BT-DNBS

Compound **7** (100 mg, 0.11 mmol) was dissolved in dichloromethane (3 mL) in a dried flask. Trifluoroacetic acid (0.6 mL) was subsequently added dropwise to the flask, and the resulting mixture was sonicated for 1 hour at room temperature. The solvent was then evaporated, and the residue underwent vacuum drying for 15 hours. Following this, the resultant material was dissolved in dichloromethane (3 mL), and N,N-diisopropylethylamine (57 µL, 0.33 mmol) was added, maintaining the reaction at 0 ℃. A mixture of dinitrobenzenesulfonyl chloride (59 mg, 0.22 mmol) and dichloromethane (2 mL) was then slowly added to the reaction system and stirred for 30 minutes. The reaction mixture was subsequently allowed to return to room temperature and stirred for an additional 16 hours. Upon completion of the reaction, as monitored by TLC (R_f=0.2, hexane: ethyl acetate = 1:1), the mixture was subjected to purification via silica gel chromatography (hexane: ethyl acetate = 2:1) employing the dry loading technique and evaporation, resulting in the isolation of compound **8** as a brown solid. (104 mg, 89%)

^{1}H NMR (500 MHz, $CDCl_3$, TMS, r.t.): δ 8.53 (dd, *J* = 8.5, 2.0 Hz, 1H), 8.49 (d, *J* = 2.0 Hz, 1H) 8.38 (d, *J* = 6.5 Hz, 2H), 8.26 (d, *J* = 9.0 Hz, 1H), 7.68 (d, *J* = 4.0 Hz, 1H), 7.67 (d, *J* = 7.5 Hz, 2H), 7.57 (d, *J* = 9.0 Hz, 2H), 7.32 (d, *J* = 4.0 Hz, 1H), 6.93 (d, *J* = 9.0 Hz, 2H), 3.54 (t, *J* = 5.0 Hz, 4H), 3.37 (t, *J* = 5.0 Hz, 4H).

^{13}C NMR (125 MHz): δ 152.24, 151.56, 150.49, 148.94, 147.54, 146.84, 137.57, 135.22, 133.16, 131.46, 127.79, 126.59, 125.32, 124.55, 120.19, 117.01, 48.66, 45.77.

ESI-Mass (m/z): $[M]^+$ calcd for $C_{43}H_{21}BF_{15}N_5O_6S_2^+$: 1063.0787; Found: 1063.0808.

Fig. S1, ^{1}H NMR of (4-(5-(4-(4-(*tert*-butoxycarbonyl)piperazin-1-yl)phenyl)thiophen-2-yl)-1λ^4-pyridin-1-yl)tris

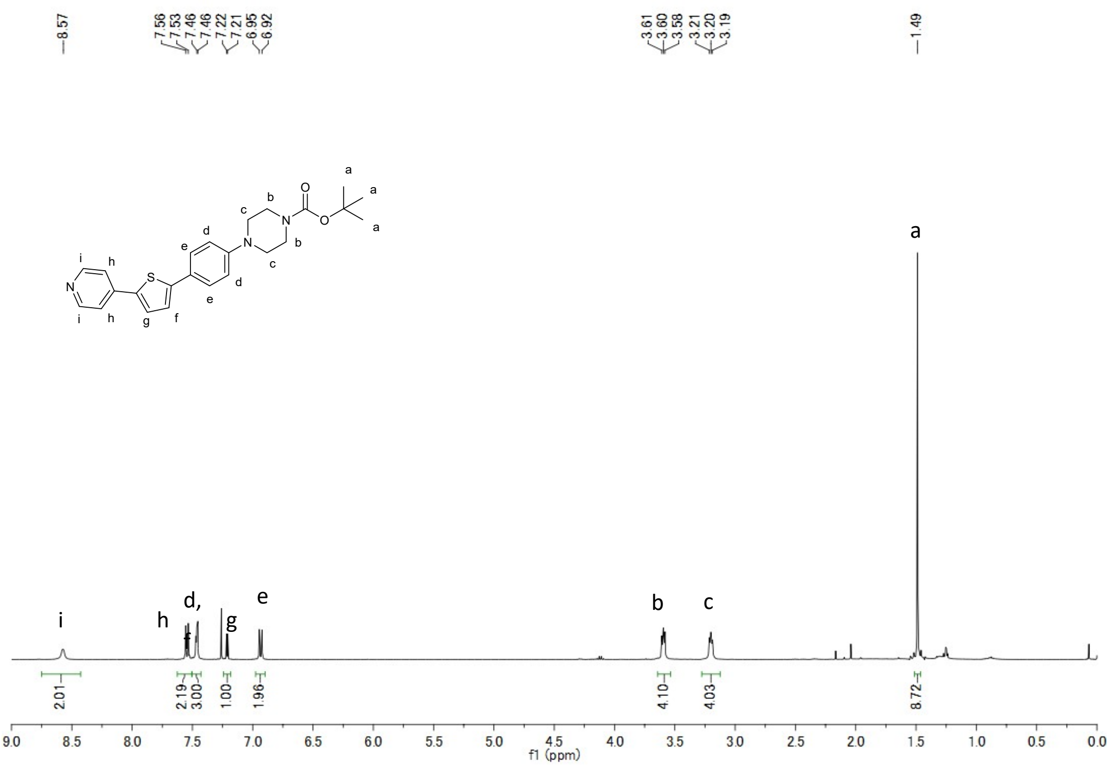

(perfluorophenyl)borate (**6**), **PT-Boc.**

Fig. S2, ^{1}H NMR of (4-(5-(4-(4-(*tert*-butoxycarbonyl)piperazin-1-yl)phenyl)thiophen-2-yl)-1λ^{4}-pyridin-1-yl)tris (perfluorophenyl)borate (**7**), **BT-Boc.**

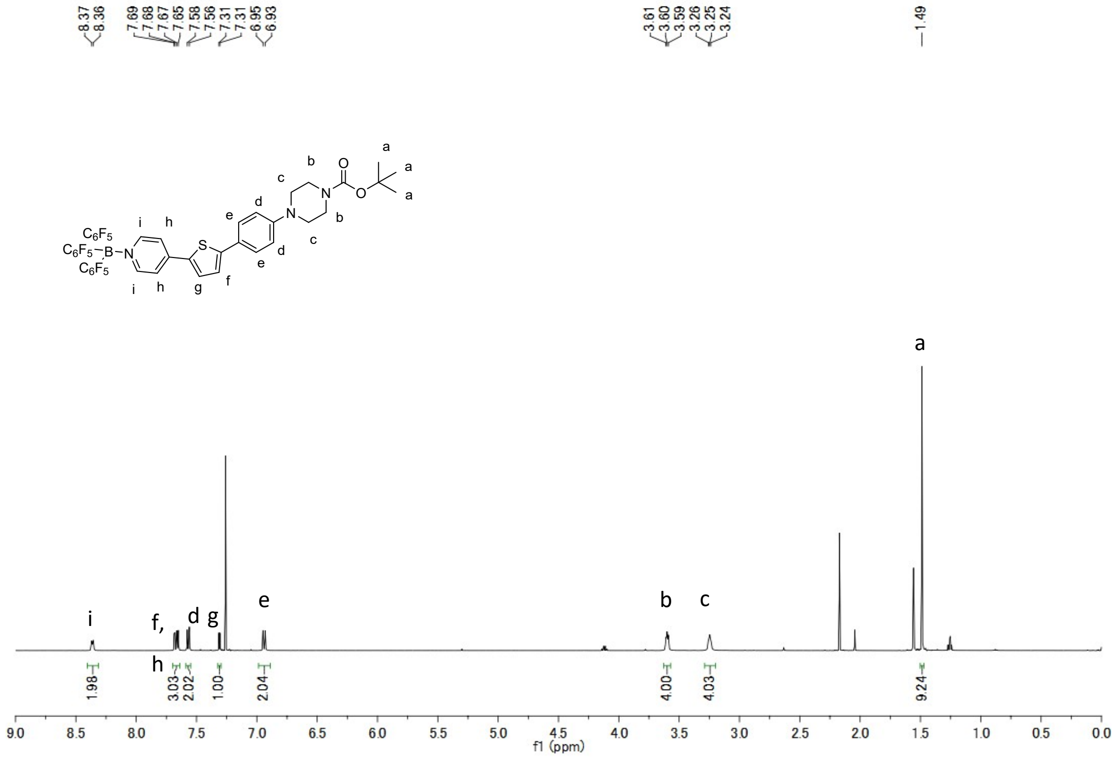

Fig. S3, ^{1}H NMR of (5-(5-(4-(4-((2,4-dinitrophenyl)sulfonyl)piperazin-1-yl)phenyl)thiophen-2-yl)-1λ^{4}-pyridin-1-yl) tris(perfluorophenyl)borate (**8**), **BT-DNBS**

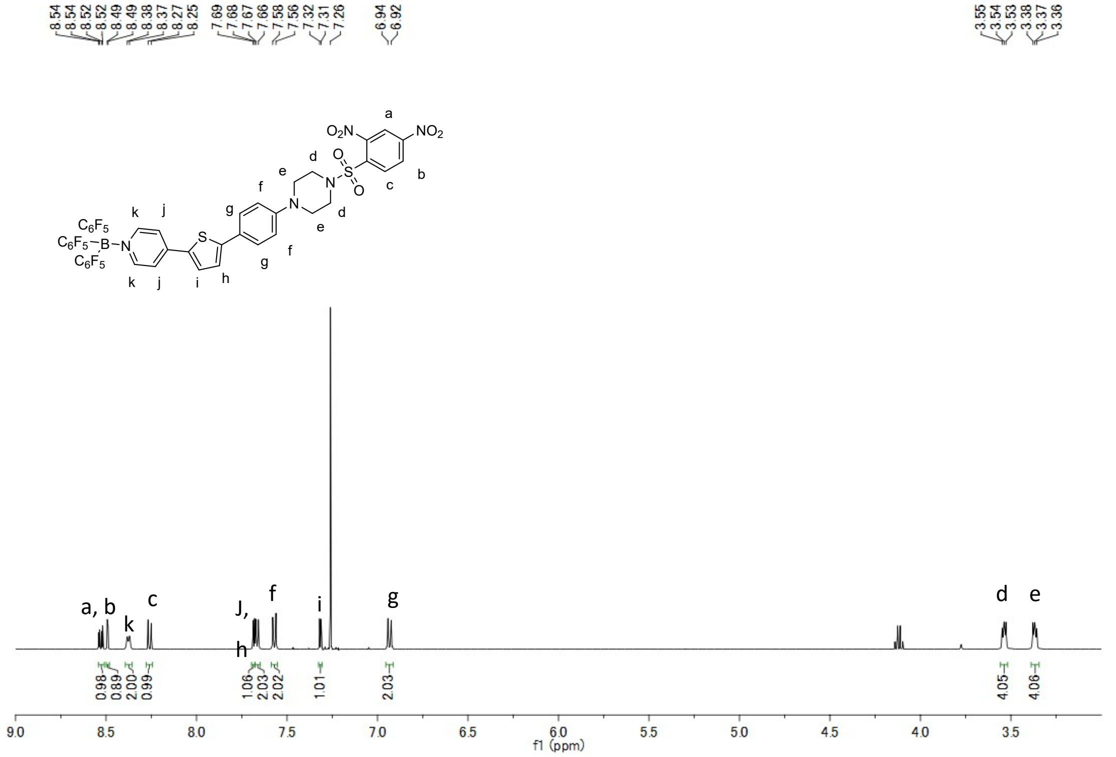

Fig. S4, ^{13}C NMR of (5-(5-(4-(4-((2,4-dinitrophenyl)sulfonyl)piperazin-1-yl)phenyl)thiophen-2-yl)-1λ^4-pyridin-1-yl) tris(perfluorophenyl)borate (**8**), **BT-DNBS**

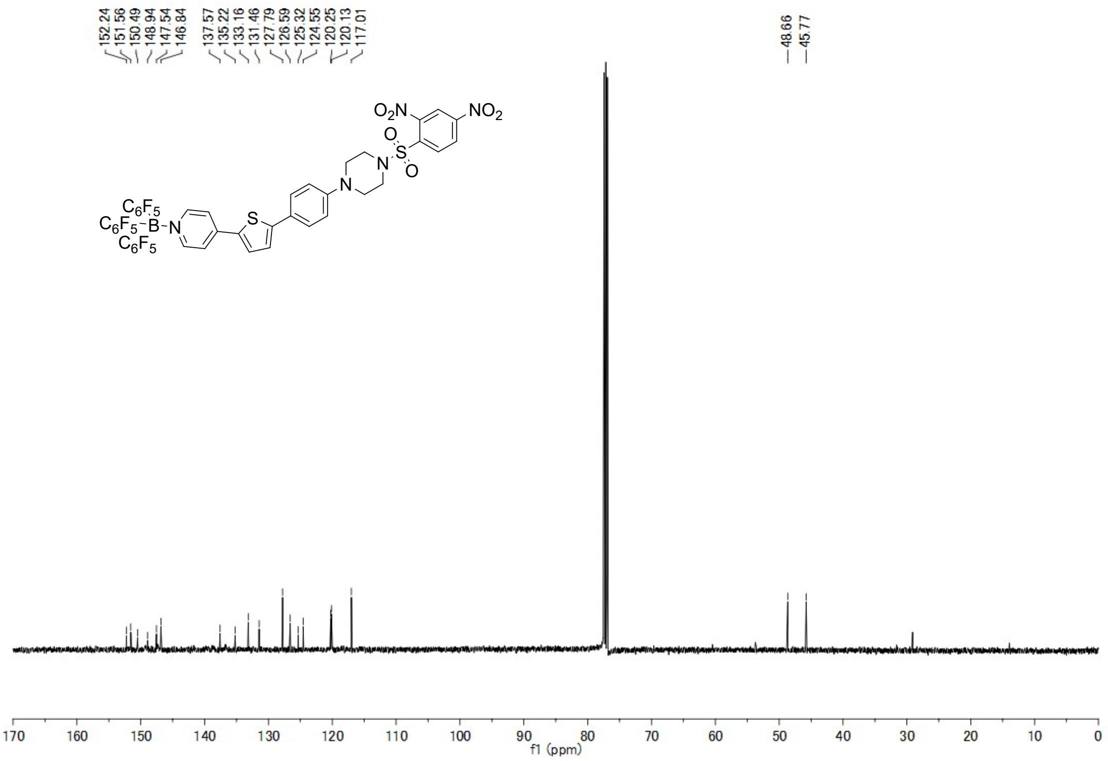

Fig. S5, ESI-Mass spectrum of (4-(5-(4-(4-(tert-butoxycarbonyl)piperazin-1-yl)phenyl)thiophen-2-yl)-1λ^4-pyridin-1 -yl)tris(perfluorophenyl)borate (**7**), **BT-Boc**

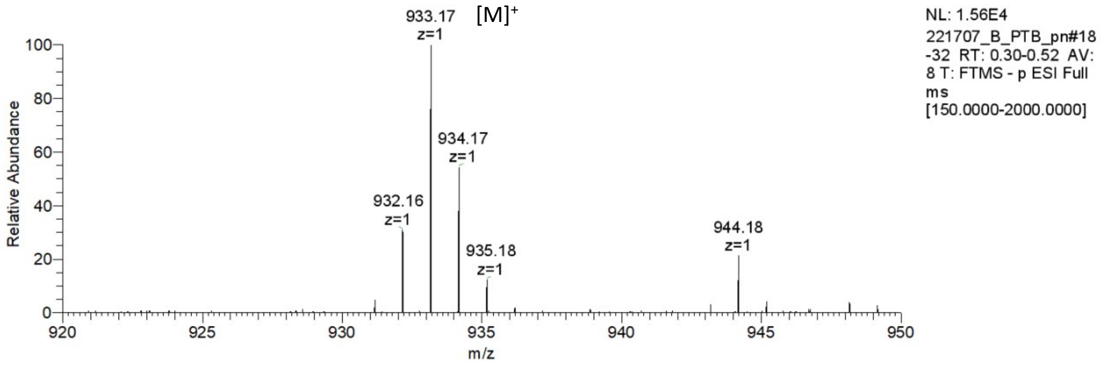

Fig. S6, ESI-Mass spectrum of (5-(5-(4-(4-((2,4-dinitrophenyl)sulfonyl)piperazin-1-yl)phenyl)thiophen-2-yl)-1λ^4 -pyridin-1-yl)tris(perfluorophenyl)borate (**8**), **BT-DNBS**

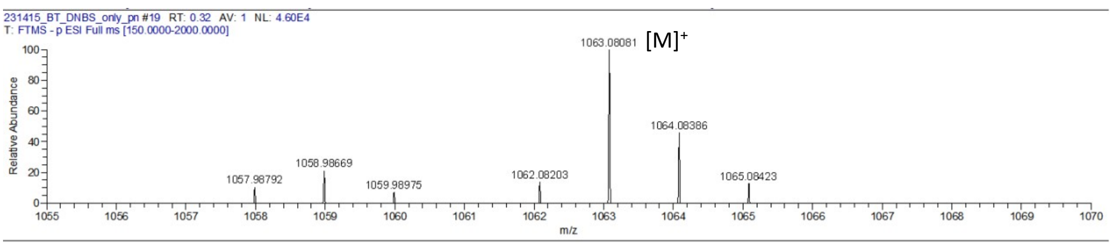

2. Spectra Analysis and Live-Cell Imaging of BT-DNBS

Instruments and Methods

Absorption spectra were measured on a JASCO V-560 spectrophotometer. Fluorescence spectra were measured by a HITACHI F-7000 fluorescence spectrometer. pH was measured by a compact pH meter LAQUATWIN-PH-11B. Cell images were acquired with IX83 microscope (Olympus, Tokyo, Japan) equipped with a BioPoint MAC 6000 filter and shutter control unit (Ludl Electronic Products, Hawthorne, NY, USA), an automated XY-stage (Applied Scientific Instrumentation, Eugene, OR, USA), a UPlanSApo 60/1.35 oil objective lens, an LDI laser light source (Chroma Technology Corp., Bellows Falls, VT, USA), and an X-Light V3 (CrestOptics, Rome, Italy) spinning-disk confocal unit. The detector used in this study was an EM-C2 electron-multiplying (EM) cooled charge-coupled device (CCD) camera iXon Ultra 888 (Oxford Instruments, Abingdon-on-Thames, UK). The excitation and emission filters adopted for this study included ZET 445/520 and ET 600/50m (Chroma Technology Corp., Bellows Falls, VT, USA). A ZT445/520 dichroic mirror (Chroma Technology Corp.) was used throughout this study. Optical density of cytotoxicity assay was measured by a BIO-RAD Benchmark Microplate Reader.

Preparation for Spectroscopic Analysis

BT-DNBS was dissolved in DMSO to prepare a 50 μM stock solution. All analytes were dissolved in PBS buffer to prepare 1 mM stock solutions, freshly prepared before each experiment. Unless otherwise noted, all spectroscopic analyses were conducted at room temperature with an incubation period of 20 minutes. The experimental conditions included **BT-DNBS** at 10 μM and analytes at 100 μM, in a DMSO/PBS buffer (9:1, v/v), with excitation at 434 nm and emission at 630 nm. Detection was calculated using the formula: Detection limit = 3**σ**/**k** (where **σ** represents the standard deviation of the blank measurements, **k** is the slop of the fluorescence intensity (**FI**) versus analytes concentration curve.

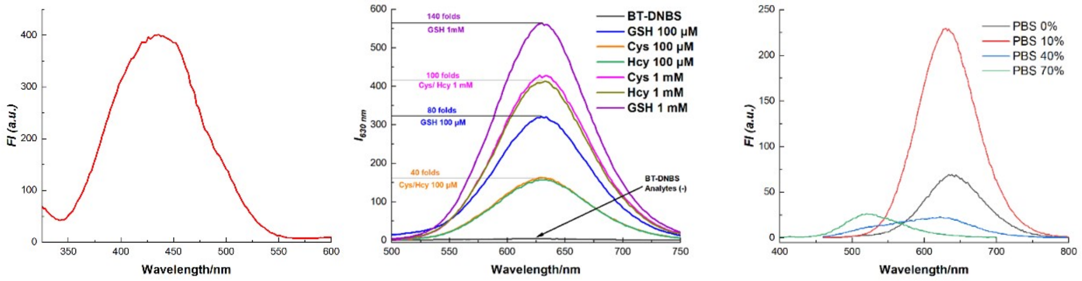

Fig. S7, Excitation spectra of **BT-NH** and fluorescence spectra of **BT-DNBS** in response to biothiols and the effect of varying PBS fractions on fluorescence intensity. left): excitation spectra of **BT-NH** in DMSO/PBS (9:1, v/v, pH 7.4). middle): fluorescence spectra of **BT-DNBS** (10 μM) in DMSO/PBS (9:1, v/v, pH 7.4) following a 20-minute incubation with biothiols, λ_{ex}=434 nm, λ_{em}=630 nm. right): fluorescence spectra of **BT-DNBS** (10 μM) in DMSO with varying PBS fractions after a 20-minute incubation with GSH (100 μM).

Table S1, Spectroscopic data of **BT-DNBS** and **BT-NH** in DMSO: PBS =9:1, v/v, pH 7.4.

Dye	$\lambda_{abs}{}^{a}$/nm	$\lambda_{ex}{}^{b}$/nm	$\lambda_{em}{}^{c}$/nm	ε^{e}/$M^{-1}cm^{-1}$	Stokes shift/cm^{-1}
BT-DNBS	434	434	629	3.5×10^{4}	7.14×10^{3}
BT-NH	436	440	630	2.4×10^{4}	7.06×10^{3}

Cell Viability Assay

a: absorption maximum. b: excitation wavelength. c: fluorescence emission maximum. e: molar extinction coefficient.

Cell Viability Assay of **BT-DNBS** was assessed using the Cell Counting Kit-8 (CCK-8) assay on A431 cells. Cells were seeded in a 96-well plate at a density of 1×10^{4} cells per well and allowed to adhere overnight. Subsequently, the cells were treated with fresh medium containing varying concentrations of **BT-DNBS** (0, 1, 10, 100 μM) and incubated for 24 hours at 37°C in a humidified atmosphere with 5% CO_2. Following the incubation period, 10 μL of CCK-8 solution was added to each well, and the plate was incubated for an additional hour. Absorbance at 450 nm was measured using a microplate reader to determine cell viability. Cell viability was calculated as a percentage relative to the untreated control cells using the following formula:

$$Cell\,Viability(\%) = \left(\frac{Absorbance\ of\ treated\ cells\ -\ Absorbance\ of\ blank}{Absorbance\ of\ control\ cells\ -\ Absorbance\ of\ blank}\right) \times 100$$

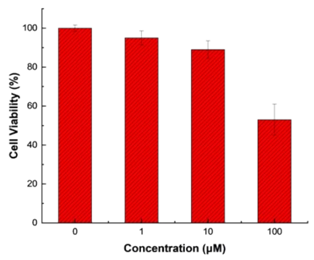

Fig. S8, Cell viability assay: A431 cells were treated with various concentrations of **BT-DNBS** (0, 1, 10, 100 μM) for 24 hours.

Live-cell imaging

A431 cells were cultured in DMEM supplemented with 10% fetal bovine serum (FBS) at 37 °C in a humidified atmosphere with 5% CO_2. Upon reaching approximately 80% confluency, the cells were treated with **BT-DNBS** (10 μM) for specified durations (60, 120, and 240 minutes). After incubation, the cells were washed three times with PBS. For the NEM group, A431 cells were pre-treated with NEM (50 μM) for 60 minutes, followed by incubation with **BT-DNBS** (10 μM) for 120 minutes. Cell images were acquired with spinning disk confocal microscopy.

Concentration Titration

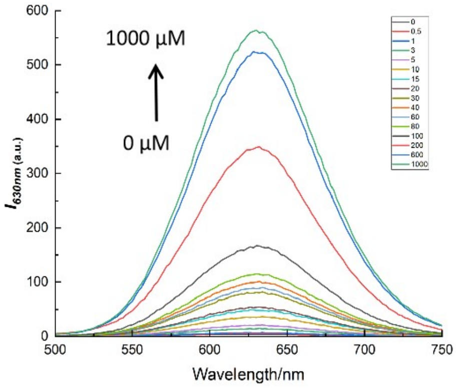

BT-DNBS+GSH

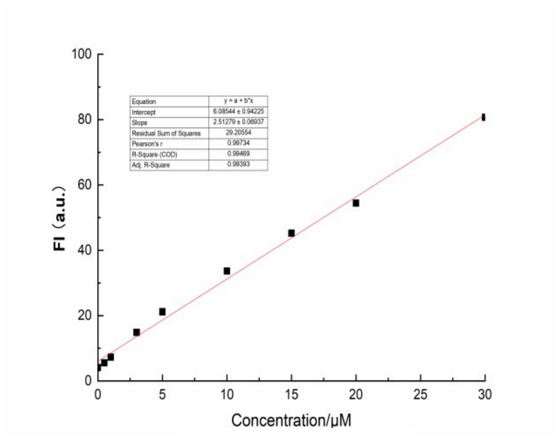

BT-DNBS+GSH

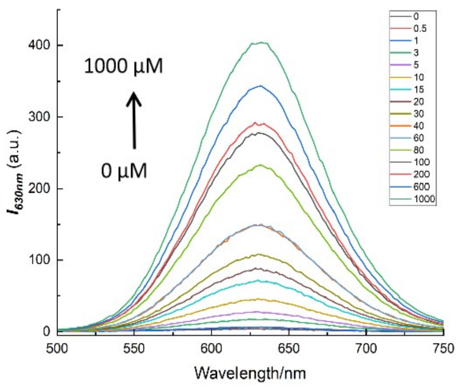

BT-DNBS+Cys

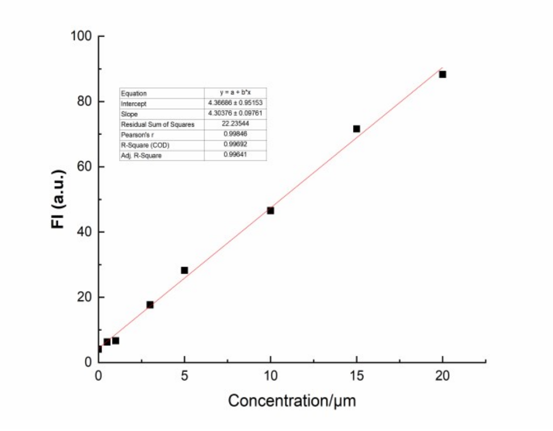

BT-DNBS+Cys

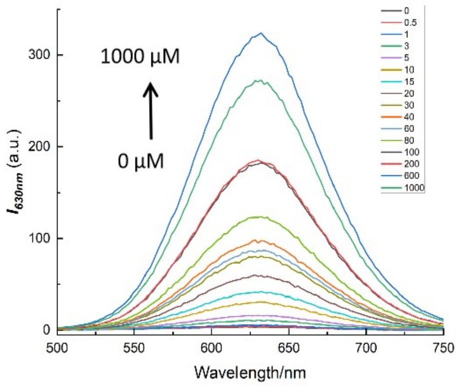

BT-DNBS+Hcy

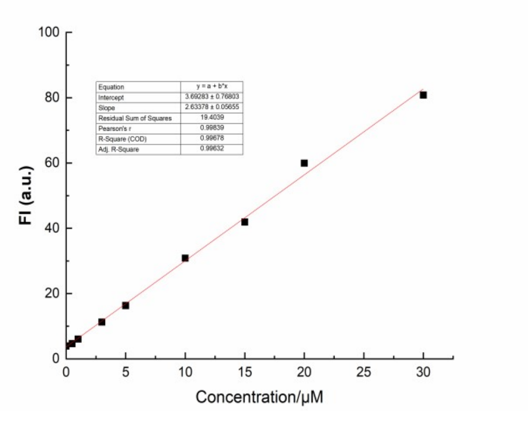

BT-DNBS+Hcy

Fig. S9, (Left) Fluorescence intensity changes of **BT-DNBS** (10 µM, DMSO/PBS=9/1, v/v) upon addition of different concentrations of GSH/Cys/Hcy in PBS, (pH=7.4). (Right) Linear relationship between fluorescence intensity and analyte concentrations at 630 nm. Incubated for 20 min, r. t., λ_{ex}=434 nm, λ_{em}=630 nm. R^2=0.99469 (GSH), 0.99692 (Cys), 0.99678 (Hcy). LOD=83 nM (GSH), 49 nM (Cys), 80 nM (Hcy).

Table S2, Reported fluorescent probes with a DNBS group in literature.

Ref	Probe	$\lambda_{ex}/\lambda_{em}$ (nm)	Stokes shift (nm)	Response time to biothiol	LOD
1	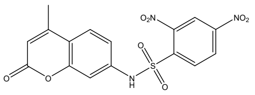	353/450	97	2 h	—
2	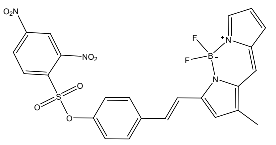	527/570	43	＞1 min	—
3	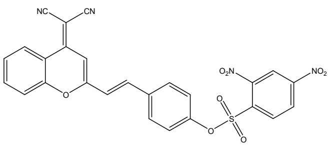	560/690	130	5 min	18 μM
4	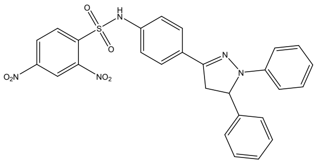	370/464	94	12 h	411 nM
5	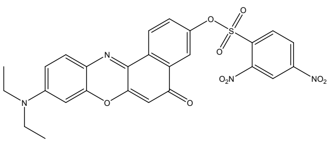	567/645	78	＞15 min	107 nM
6	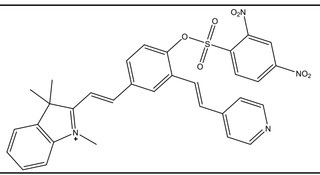	545/604	59	8 min	134 nM
7	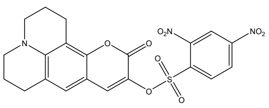	395/517	122	24 min	14.5 nm
8	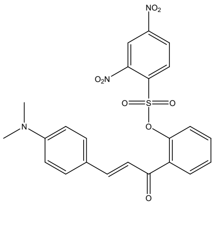	460/600	140	15 min	227 nm
This study	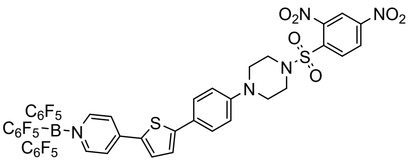	434/630	196	20 min	83 nm

References

1. M. Wei, P. Yin, Y. Shen, L. Zhang, J. Deng, S. Xue, H. Li, B. Guo, Y. Zhang, S. Yao, A new turn-on fluorescent probe for selective detection of glutathione and cysteine in living cells, *Chem. Commun.,* 2013, **49**, 4640−2.
2. X. Li, S. Qian, Q. He, B. Yang, J. Li, Y. Hu, Design and synthesis of a highly selective fluorescent turn-on probe for thiol bioimaging in living cells, *Org. Biomol. Chem*., 2010, **8**, 3627–30.
3. M. Li, X. Wu, Y. Wang, Y. Li, W. Zhu, T. D. James, A near-infrared colorimetric fluorescent chemodosimeter for the detection of glutathione in living cells, *Chem. Commun*., 2014, **50**, 1751–3.
4. S. Q. Wang, Q. H. Wu, H. Y. Wang, X. X. Zheng, S. L. Shen, Y. R. Zhang, J. Y. Miao, B. X. Zhao, A novel pyrazoline-based selective fluorescent probe for detecting reduced glutathione and its application in living cells and serum, *Analyst*, 2013, **138**, 7169−74.
5. X. Rong, Z. Y. Xu, J. W. Yan, Z. Z. Meng, B. Zhu, L. Zhang, Nile-Red-Based Fluorescence Probe for Selective Detection of Biothiols, Computational Study, and Application in Cell Imaging, *Molecules*, 2020, **25**, 4718.
6. Y. Zhu, H. Pan, Y. Song, C. Jing, J. A. Gan, J. Zhang, Mitochondria-targeted fluorescent probe for rapid detection of thiols and its application in bioimaging, *Dyes and Pigments*, 2021, **191**, 109376.
7. X. Wang, D. Zhang, A novel reaction-based fluorescent turn-on probe for biothiols and its application in cell imaging, *Sensors and Actuators B: Chemical*, 2017, **241**, 327-334.
8. F. Dai, M. Z. Yang, T. Wang, C. Wang, An ESIPT coupled AIE fluorescent probe for biothiols detection and imaging based on a chalcone fluorophore, *Dyes and Pigments*, 2020, **183**, 108627.
